# Confounding variables impacting the association between duration of veno-arterial extracorporeal life support and mortality

**DOI:** 10.1186/s13054-017-1773-3

**Published:** 2017-08-01

**Authors:** Jesse Kiefer, Robert E. Freundlich

**Affiliations:** 1 Department of Anesthesiology, Division of Anesthesia Critical Care, Vanderbilt University Medical Center, Nashville, TN USA; 2 Department of Anesthesiology, Division of Anesthesia Critical Care, Vanderbilt University Medical Center, 1211 21st Avenue South, MAB Suite 526, Nashville, TN 37232 USA

## Main Text

We read with great interest the analysis by Smith et al. [1] on the correlation between duration of veno-arterial extracorporeal life support (VA-ECMO) and outcomes, using data from the extracorporeal life support organization (ELSO). We note their finding that decannulation at day four of VA-ECMO is associated with the highest percentage of patients surviving.

While this is the largest analysis of VA-ECMO duration to date, it is limited by use of a retrospective database that appears to offer inadequate detail on cannulation strategies. As with any multivariate logistic regression performed on an observational dataset, the results need to be interpreted in the context of availability of adequate data on confounding variables. We would argue that cannulation site, whether central cannulation or peripheral cannulation, is a significant confounding variable when assessing survival on VA-ECMO [2]. Similarly, strategies to decompress the left ventricle are critical in optimizing survival on VA-ECMO. Techniques such as left ventricular venting, intra-aortic balloon pump placement, or the Impella ® (Abiomed, Danvers, MA) are commonly employed in this setting and should be accounted for in the statistical analysis [3].

We would encourage the ELSO group to work to improve data collection around the use of these techniques, to facilitate improved statistical analysis and a better understanding of the implications of placing patients on VA-ECMO for prolonged periods of time.

## Abbreviations

ELSO: Extracorporeal life support organization
VA-ECMO: Veno-arterial extracorporeal life support
