# Supplementary material for: Confounding variables impacting the association between duration of veno-arterial extracorporeal life support and mortality
Source: Crit Care. 2017 Aug 1;21:203. doi: 10.1186/s13054-017-1773-3 (PMC5539615; doi:10.1186/s13054-017-1773-3)
Supplement: Related texts

# Reply: Authors’ response

**Authors:** Myles Smith, Daniel Brodie, Ravi Thiagarajan, Hergen Buscher

**Affiliations:** 3 St Vincent’s Hospital, Department of Intensive Care Medicine, Sydney, Australia; 4 Extracorporeal Life Support Organization (ELSO), Ann Arbor, USA; 5 0000 0004 4902 0432 grid.1005.4 University of New South Wales, Sydney, Australia; 6 0000000419368729 grid.21729.3f Columbia University Medical Center/NewYork-Presbyterian Hospital, New York, USA

We thank Drs. Kiefer and Freundlich, who correctly comment that any retrospective analysis of a database, such as the ELSO registry, is limited by the persistence of confounding variables. We agree that cannulation strategy, as well as other techniques mentioned, such as left ventricular venting and intra-aortic balloon pump placement, may have an impact on both treatment duration and survival.

While the ELSO registry allows for collection of many disease-, patient-, and treatment-related variables [4], we had to focus on key covariates that were anticipated to occur commonly and influence duration and survival. While cannulation site data may be available from the data collected, use of other devices to “vent” the left ventricle or facilitate weaning is not (other than a statement on whether the patient was “converted to other support”). There is a challenge for data collection to be both complete and standardized. Currently, there is no agreed upon standard of care for the use of venting, new approaches to venting are reported frequently, and new devices are being introduced. We agree that this should be a focus of further research, in particular to delineate the role of left ventricular decompression for which a variety of strategies are reported with only few reports to date on their effect on duration and outcome [5, 6].
